# Bingwen Zou (Ping-wen Tsou): pioneer of agricultural higher education in China

**DOI:** 10.1007/s13238-021-00830-1

**Published:** 2021-03-04

**Authors:** Yuanchao Wang

**Affiliations:** College of Plant Protection, Nanjing Agriculture University, Nanjing, 210095 China

**Figure 1:**
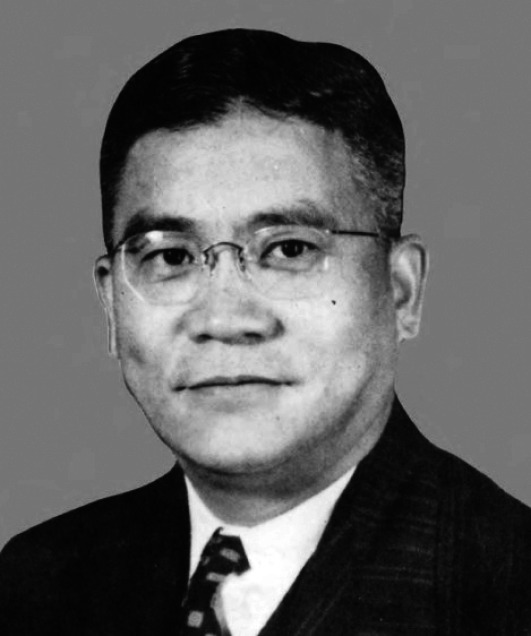
Bingwen Zou (1893.12–1985.6). (from Zou Bingwen Memorial Collection (《邹秉文纪念集》))

Bingwen Zou, a renowed agronomist and educator, is one of the founders of agricultural higher education in China (Fig. 1) (Liu and Liu, 1993). He is committed to set up modern agriculture education in China and supervised many students who later became well-known experts in Agriculture, including Shanbao Jin, Zefang Feng and Zhonglin Zou. Bingwen Zou made great contributions to the development of modern agriculture science in China, and was honored as one of the “Three Outstanding Scholars in Southeast” by premier Enlai Zhou.

Bingwen Zou was born in 1893 in Jingsu Province. He received high school education in the United States from 1910 to 1912 and continued higher education in Cornell University. During his study in Cornell University, Bingwen Zou was firstly enrolled into Mechanical Engineering. Soon afterwards, he realized that although China claims to established the country on agriculture, but the development of agriculture was declining gradually, and suffered from serious plant diseases. Therefore, he quitted from Mechanical Engineering to study Agriculture, majoring in Plant Pathology. In 1915, Bingwen Zou, together with his alumni in Cornell, initiated the first comprehensive Chinese scientific association–The Science Society of China, and compiled one of the most influential journal—Kexue (Science) monthly. Bingwen Zou is also one of the founders of the Chinese Society of Agriculture founded in 1917.

Bingwen Zou graduated from Cornell University in 1915 and returned to China in 1916. He was invited by Rui Silou, the director of the Department of Agriculture and Forestry at Jinling University, to teach plant pathology and botany courses at Jinling University. He emphasized the importance of quarantine and gave the earliest suggestion of plant quarantine in China in 1916. Bingwen Zou was the first person to teach plant pathology in China and made great efforts to become an excellent plant pathologist. With the deeper understanding of the problems in Chinese agricultural society, the deeper consideration to develop higher agricultural education in China. His efforts are not confined to the development of the plant pathology, but in the whole construction of agricultural science by reforming and promoting Chinese agricultural education (Geng, 2015).

From 1917, Bingwen Zou became director of Agricultural Science in Nanjing Higher Normal School. In November 1920, Bingwen Zou, together with Yuanpei Cai, Jian Zhang and Yanpei Huang, proposed the establishment of the Southeast University in Nanjing. Several departments of Nanjing Higher Normal School were transferred to Southeast University. In 1921, Southeast University was officially established with three branches: Engineering, Commerce and Agriculture. Bingwen Zou was the first director of the Agriculture department. He invited a number of famous professors such as Xiansu Hu, Chongshu Qian, Songzhou Yuan, Enlin Sun and Jubo Zhang to teach at the University. He questioned and refused to use Japanese, European and American textbooks as teaching materials for Agricultural science in Universities. He took the lead to compile the first college textbook of botany in Chinese—*Advanced Botany*—in 1923 (Zou et al., 1923). In addition, Bingwen Zou pointed out that lack of field practice was a major problem for Chinese agricultural education. He reformed the traditional teaching mode in the University from text-book based knowledge learning to a mode combining teaching with research and practice.

Bingwen Zou initiated the establishment of the Cotton Improvement and Promotion Committee under the Agricultural department of Southeast University, and took the lead in holding the Summer Cotton Planting Workshops in Southeast University. Bingwen Zou also initiated the establishment of Jiangsu Insect Bureau in 1922 to carry out research of insect and control of cotton, rice pests and locusts, which set up a new trend to scientifically control insect in China. He trained a group of renowed entomologists such as Zhonglin Zou, and opened the prelude to modern agricultural insect research in China.

By the time he left the director position at Southeast University in 1927, the agriculture science had been well developed, with seven departments containing Agronomy, Horticulture, Plant diseases and insect pests, Animal husbandry, Agricultural chemistry, Biology and Sericulture, and nine research stations for rice, sericulture, horticulture and cotton in Jiangsu, Henan, Hubei and Hebei provinces, making the Agricultural department of Southeast University the center of agricultural science in China at that time.

**Figure 2:**
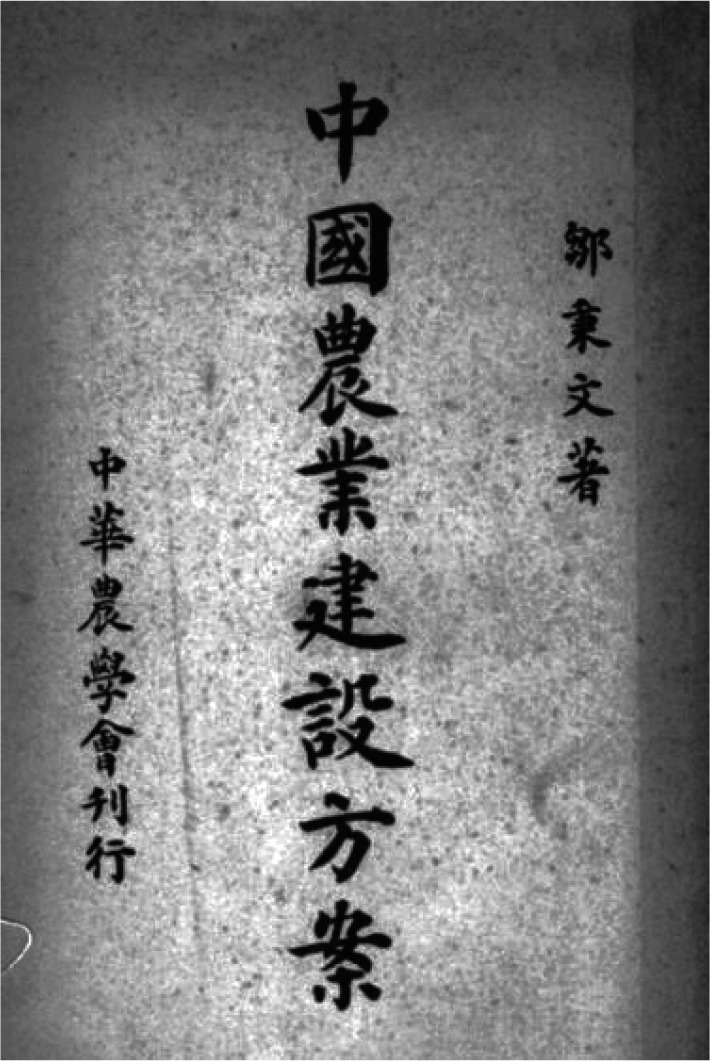
Chinese Agricultural Construction Plan raised by Bingwen Zou. (from Chinese Agricultural Construction Plan《中国农业建设方案》)

Bingwen Zou raised the *Chinese Agricultural Construction Plan* in 1945 by virtue of his advanced scientific ideas and the practical problems in Chinese agriculture, which attracted extensive attention in China and abroad (Zou, 1945). Six months after the book was published, the University of Michigan awarded Bingwen Zou an honorary doctorate in 1946 in recognition of his contributions to Chinese agricultural development (Fig. 2).

From 1943 to 1947, during Bingwen Zou served as the agricultural representative of the Government in the United States. He also served as the chief editor of the monthly magazine—China Agriculture (in English), where he openly discussed and collected suggestions on the development of Chinese agriculture. Moreover, he was engaged in persuading universities/institutions of the United States to provide scholarships and opportunities for agricultural graduate students in China. He obtained the opportunities for up to 200 graduate students for internships in universities/institutions in the United States. As the co-founder of early agricultural higher education in China, Bingwen Zou wrote the first monograph on agricultural education in China, *Agricultural Education in China* (Zou, 1923). This is based on his experience of teaching Agriculture in Jinling University and Southeast University, extensively investigating the advantages and disadvantages of agricultural education throughout the country, and referring to the precedents, such as Japan, the United States, Denmark and other countries. He pointed out a key to improve agricultural education is recruiting specialized teachers in agriculture. He mentioned that Agriculture field is so complicated that the experts can only be good at one or two aspects. The experts are often named as crop specialist, horticulturist, animal husbandry specialist, plant disease specialist, and so on, but not agricultural expert. Bingwen Zou emphasized that an expert must have expertise and be able to solve practical problems.

In 1994, following Bingwen Zou’s legacy, his family donated all his savings, to set up “Zou Bingwen Scholarship” in Nanjing Agricultural University to support talent undergraduate students. Throughout his life, Bingwen Zou devoted himself to promote the development of Agriculture in China and serve the country.
